# The evolutionary consequences for seawater performance and its hormonal control when anadromous Atlantic salmon become landlocked

**DOI:** 10.1038/s41598-018-37608-1

**Published:** 2019-01-30

**Authors:** Stephen D. McCormick, Amy M. Regish, William R. Ardren, Björn Thrandur Björnsson, Nicholas J. Bernier

**Affiliations:** 1 U.S. Geological Survey, Leetown Science Center, Conte Anadromous Fish Research Laboratory, Turners Falls, MA 01376 USA; 2 U.S. Fish and Wildlife Service, Lake Champlain Fish and Wildlife Conservation Office, 11 Lincoln Street, Essex Junction, VT 05452 USA; 3 Fish Endocrinology Laboratory, Department of Biological and Environmental Sciences, University of Gothenburg, S40530 Gothenburg, Sweden; 4 Department of Integrative Biology, University of Guelph, Ontario, N1G 2W1 Canada

**Keywords:** Metabolism, Neurophysiology

## Abstract

Populations of anadromous fish have become landlocked in relatively recent geological history (<14,000 years), but the evolutionary impacts of this altered lifecycle on traits underlying seawater performance have not been established. In order to examine the effects of relaxed selection on seawater traits, anadromous and landlocked Atlantic salmon were reared under identical conditions and examined for differences in seawater performance and its underlying physiological and endocrine control during the time of spring downstream migration. Salinity tolerance, survival and initial growth in seawater were greater in anadromous than in landlocked salmon. Abundance of the seawater isoform of gill Na^+^/K^+^-ATPase increased in spring in both strains but was greater in anadromous salmon. Hormones associated with seawater acclimation (adrenocorticotropic hormone, cortisol and growth hormone) increased in spring in both strains but were higher in anadromous salmon, whereas plasma thyroid hormones did not differ. Hypothalamic urotensin I mRNA levels also increased in spring and were higher in the anadromous strain. The results provide evidence that salinity tolerance and associated physiological traits are regulated by seasonal stimulation of the hypothalamic-pituitary-interrenal axis, and that relaxed selection on seawater entry traits has decreased this stimulation in landlocked salmon.

## Introduction

Hormones control critical life history traits including migration, growth, development and reproduction in all vertebrates^1–3^. These endocrine processes are critical to the survival of individuals and likely to be under strong selection pressure. In spite of the recognition of the importance of endocrine traits, however, there are relatively few examples of how they have been shaped by evolution or the speed at which this might occur. Pigliucci has termed hormones the “unsung heroes of the nature-nurture field of study”^4^ in part due to the relatively few clear demonstrations of how the hormonal control of life history traits has been shaped by evolution. This deficit may stem from the pleiotrophic nature of hormonal regulation and the complexity and interaction of endocrine pathways^5^.

The complex life history of anadromous Atlantic salmon includes downstream migration and ocean entry during the smolt stage, after which fish spend 1–3 years in the ocean before returning to freshwater (FW) to spawn. Smolts develop increased hypo-osmoregulatory capacity, which is vital for seawater survival, through development of specialized branchial ionocytes with the capacity to secrete sodium and chloride^6^. Salmonids possess salinity-specific isoforms of the enzyme Na^+^/K^+^-ATPase (NKA) that drive ion transport in ionocytes^7,8^, and recent evidence indicates that increased abundance of the NKAα1b isoform in gill ionocytes during smolting underlies elevated salinity tolerance^9^.

A number of hormones increase during smolt development, bodily response which has been referred to by Bern as a”pan-hyperendocrine state”^10^. Increased growth hormone (GH) and cortisol levels are both strongly linked to increased salinity tolerance^11^. Thyroid hormones (THs) also increase during smolting, and are likely to be important in morphological changes and the process of imprinting. While there have been a large number of studies examining changes in circulating hormones during smolting, there have been relatively few investigations of changes in the pituitary and brain that are the upstream regulators of plasma hormone levels^11^.

Landlocked populations that use lakes rather than the ocean for post-smolt growth offer an opportunity to examine the rapid evolution of life history traits associated with seawater (SW) entry. There are a number of Atlantic salmon populations in North America and Europe that have anadromous origins, all of which appear to have been independently landlocked due to geological changes after the most recent glacial retreat ~15,000 years ago^12–14^. These populations undergo downstream and in-lake migration at a similar size and time of year as anadromous salmon^15^, but as the whole lifecycle takes place in FW, they will have experienced relaxed selection on traits associated with salinity tolerance, but not on other aspects of smolting such as changes in morphology and the capacity to migrate. While comparisons have been made between landlocked and anadromous populations^8,16,17^, to our knowledge no complete common garden experiments have examined the neuroendocrine control of smolt development. We carried out the present study to test the prediction that relaxed selection on ocean entry in landlocked salmon would result in reductions in physiological and neuroendocrine traits associated with SW tolerance, whereas other endocrine aspects of smolt development would not be altered. We used a common garden experimental design to compare landlocked and anadromous Atlantic salmon and examined changes in FW over time during normal smolt development in spring, with monthly SW challenges and a single long-term SW survival and growth trial. We examined physiological traits that underlie increased salinity tolerance including gill NKA activity and abundance of the ‘freshwater’ (NKAα1a) and ‘seawater’ (NKAα1b) isoforms of this salt transporting enzyme which have not previously been compared in landlocked and anadromous salmon. We measured circulating levels of growth hormone, insulin-like growth factor I (IGF-I), cortisol, adrenocorticotropic hormone (ACTH), and THs to determine how these have been shaped by landlocking. In order to compare the upstream regulators of circulating cortisol, we examined brain (hypothalamus and prepotic area (POA)) mRNA levels of corticotropin-releasing factor (*crf*) and urotensin I (*uts1*), the major releasing factors of ACTH, the three major isoforms of pro-opiomelanocortin in the pituitary (*pomc-a1*, *pomc-a2 and pomc-b*) which is the precursor for ACTH, and prohormone convertases (*pc1* and *pc2*) which convert POMC to ACTH. These components of the hypothalamic-pituitary-interrenal axis have not previously been measured during smolt development of salmonids.

## Results

### Morphometry

**Figure 1:**
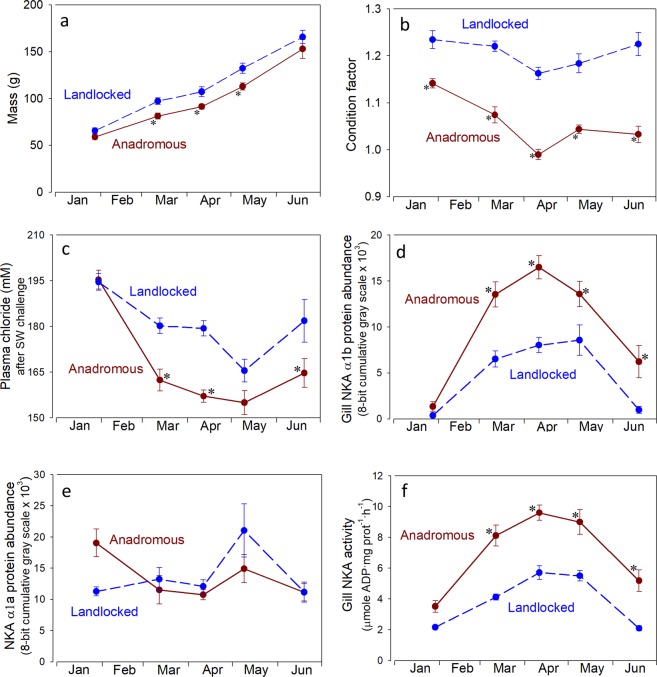
Whole body mass (**a**), condition factor (100 · (mass^.^ length^−3^)) (**b**), plasma chloride (**c**), abundance of gill NKAα1b (**d**) and NKAα1a (**e**) and NKA activity (**f**) in juvenile anadromous and landlocked juvenile Atlantic salmon in freshwater (**a**,**b**,**d**–**f**) or after 24 h seawater challenge (c; 35 ppt). Values are mean ± standard error of 10–12 individuals. For plasma chloride, gill NKA activity and NKAα1b there was a significant effect of time (p < 0.0001), strain (p < 0.0001) and a significant interaction (p < 0.02). For gill NKAα1a there was a significant effect of time (p = 0.0036), no effect of strain (p = 0.82) but a significant interaction (p = 0.019). Asterisk indicates significant difference from the landlocked strain at the same time period (p < 0.05, Neuman-Keuls test).

Juvenile landlocked salmon were slightly larger and more robust than the anadromous salmon (Fig. 1a,b). Condition factor decreased in both groups during spring as is typical for smolts (Fig. 1b), but both the proportion and absolute decrease was greater in anadromous salmon. Increased silvering and darkening of fin margin occurred in spring in both groups, but was subjectively greater in the anadromous salmon.

### Seawater Performance

Direct transfer of fish to 35 ppt SW (SW challenge test) in late January led to high plasma chloride levels (Fig. 1c), typical for juveniles that have not yet begun smolt development. Lower levels of plasma chloride (indicative of increased hypo-osmoregulatory capacity) were seen in each strain from March through May, but with lower levels in the anadromous strain throughout this period. Both strains showed a partial loss of salinity tolerance in June.

Growth in 32 ppt SW was assessed during three sequential 10-day periods from April 19^th^. During the first ten days, both groups lost weight, but there was significantly more weight loss for the landlocked fish (p = 0.008; Table 1). This effect was unlikely due to greater dehydration of the landlocked fish, as muscle moisture content of fish in 32 ppt for 8 days did not differ between the two strains (p = 0.62). During the following growth periods (10–30 days), growth was positive for both strains, and there was no significant difference between the strains. During the first half of the SW growth trial, mortality was 17% (5 fish) for the landlocked strain, while no anadromous fish died. There were no mortalities in either group during the second half of the SW growth trial.

**Table 1:** Specific growth rate in mass (% d^−1^) of anadromous and landlocked Atlantic salmon following exposure to 32 ppt for 30 days.

Strain	Day 1–10	Day 11–20	Day 21–30
Anadromous	−0.12* ± 0.04	0.55 ± 0.06	0.70 ± 0.05
Landlocked	−0.43 ± 0.09	0.62 ± 0.05	0.70 ± 0.05

Fish were individually tagged and mass measured every ten days. There was a significant effect of time (p < 0.0001), no effect of strain (p = 0.47) and a significant interaction (p = 0.024). Asterisk indicates significant difference from the landlocked strain at the same time period.

### Na^+^/K^+^-ATPase

Abundance of the NKAα1b protein isoform in the gill was initially low in January and then increased progressively in each strain (Fig. 1d). Peak levels, which came slightly earlier in the anadromous strain, were 2-fold higher for the anadromous strain compared to the landlocked strain. Both strains showed substantial decreases of gill NKAα1b in June. In contrast, gill NKAα1a abundance was relatively high in both strains in January and though somewhat variable, stayed relatively constant throughout the spring and early summer (Fig. 1e; p = 0.82).

Gill NKA activity was low in both strains in January and increased progressively in each through May, followed by a decrease in June (Fig. 1f). Peak levels were 2-fold higher for the anadromous strain than the landlocked strain. The pattern of gill NKA activity and gill NKAα1b abundance were strikingly similar, and both were similar to the increase and subsequent loss of salinity tolerance.

### Endocrine changes

**Figure 2:**
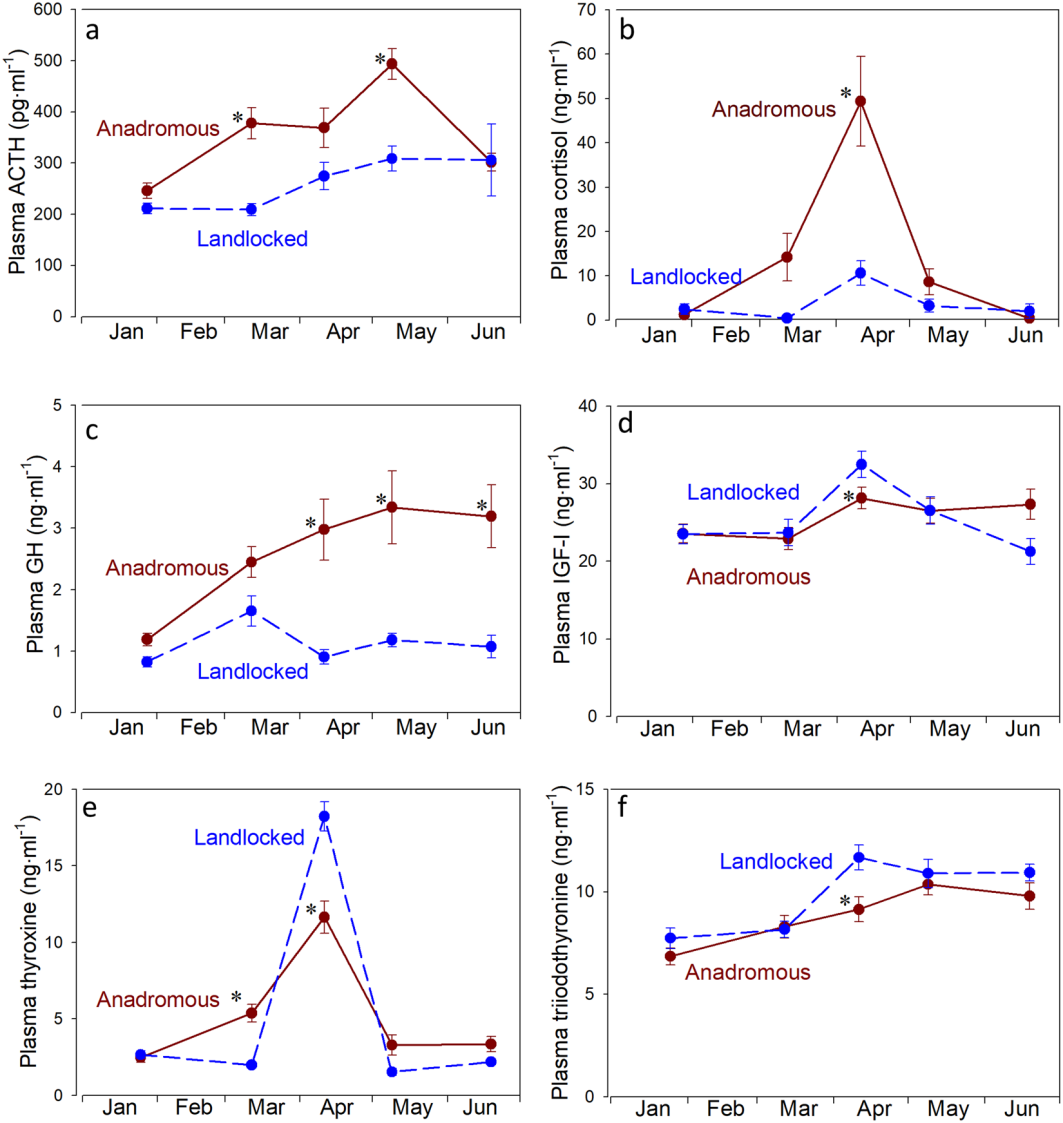
Plasma ACTH (**a**), cortisol (**b**), GH (**c**), IGF-I (**d**), thyroxine (T_4_; **e**) and triiodothyronine (T_3_; **f**) levels in juvenile anadromous and landlocked Atlantic salmon in freshwater. Values are mean ± standard error of 10–12 individuals. For plasma cortisol, ACTH and GH levels there was a significant effect of time (p < 0.0001), strain (p < 0.0001) and a significant interaction (p < 0.02). For plasma IGF-I and T_4_ levels there was a significant effect of time (p < 0.0001), no effect of strain (p > 0.8) but a significant interaction (p < 0.026). For plasma T_3_ there was a significant effect of time (p < 0.0001) and strain (p = 0.005) and no significant interaction (p = 0.15). Asterisk indicates significant difference from the landlocked strain at the same time period (p < 0.05, Neuman-Keuls test).

Plasma ACTH levels were low in both strains in January (Fig. 2a). A significant increase was observed in the anadromous strain in March and peak levels were reached in May followed by a decline in June. Plasma ACTH levels in the landlocked strain increased slightly in the spring, but peak levels were 40% lower than in the anadromous strain.

Plasma cortisol levels were low in each strain in January (Fig. 2b). There was an increase in plasma cortisol in anadromous salmon in March, peak levels occurred in April followed by declines in May and June. There was a similar pattern in landlocked salmon, but peak levels of plasma cortisol were 5-fold higher in the anadromous strain.

Plasma GH levels were lowest in January in both strains (Fig. 2c). In anadromous salmon, plasma GH rose progressively to peak levels in May that remained elevated in June. In landlocked salmon, plasma GH increased slightly in March, but then returned to low levels in April and remained low in May and June. When GH was at its peak levels in the anadromous salmon, they were 3-fold higher than the landlocked salmon. Plasma IGF-I levels were lowest in January and rose slightly in April, but did not show substantial difference between the two strains (Fig. 2d).

Plasma T_4_ levels were low in January and increased in March in anadromous salmon, but both strains had peak levels in April which were slightly higher in the landlocked strain (Fig. 2e). Plasma T_3_ levels were low in January and increased progressively through June in both strains but were slightly higher in the landlocked strain in April (Fig. 2f).

### Transcriptional changes

**Figure 3:**
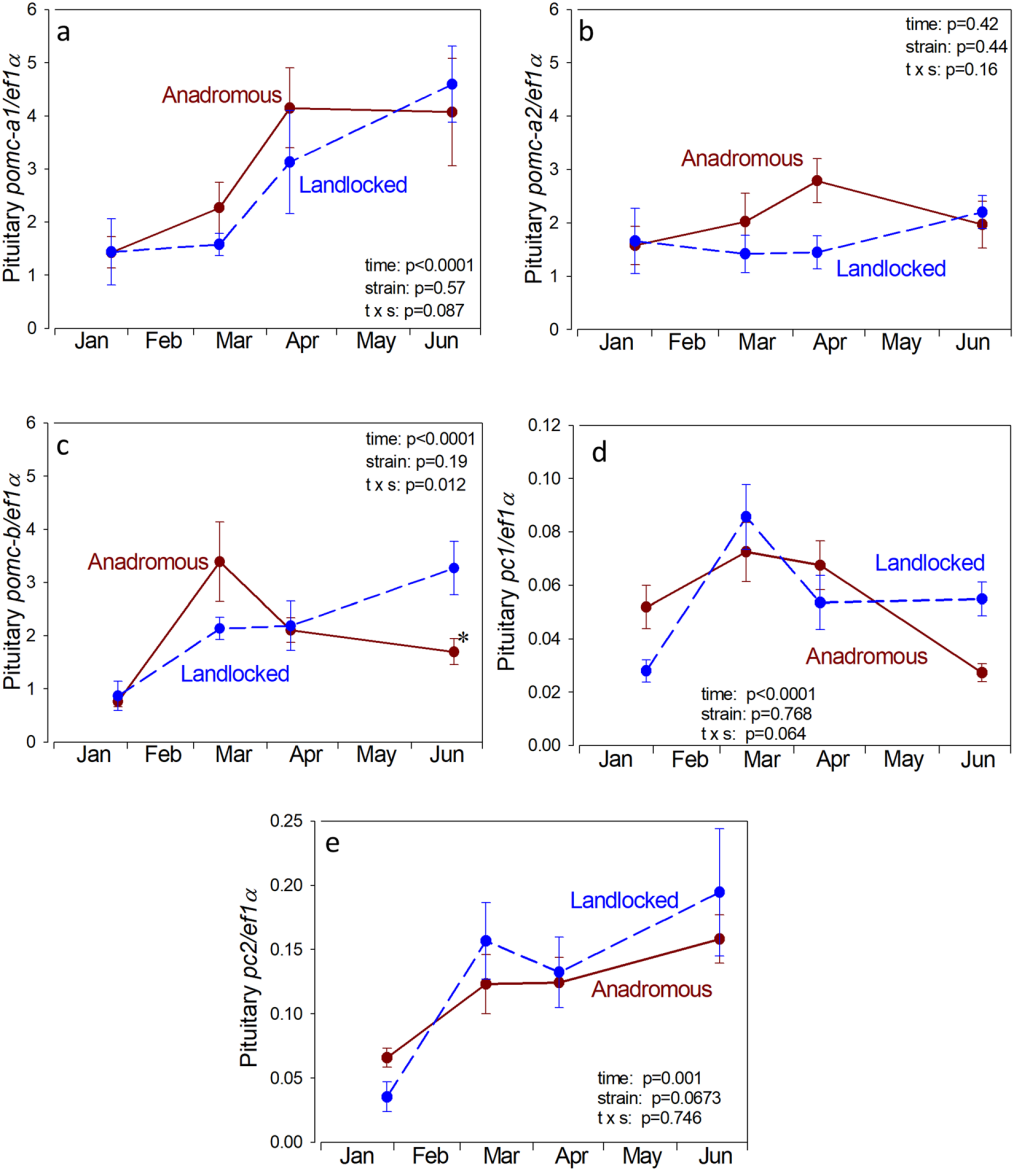
Pituitary mRNA levels for the 3 known isoforms of pro-opiomelanocortin (POMC; the precursor of ACTH), *pomc-a1* (**a**), *pomc-a2* (**b**) and *pomc-b* (**c**), and *pc1* (**d**) and *pc2 *(**e**) mRNA levels in juvenile anadromous and landlocked Atlantic salmon in freshwater. Values are relative to the housekeeping gene *ef1α* and are the mean ± standard error of 6–9 individuals. Results of two-way ANOVA are noted in the figure. Asterisk indicates significant difference from the landlocked strain at the same time period (p < 0.05, Neuman-Keuls test).

Pituitary *pomc-a1* and *pomc-a2* mRNA levels increased progressively from January through June and although they tended to be higher in the anadromous salmon, there was no statistically significant difference between strains (Fig. 3a,b). Pituitary *pomc-b* mRNA levels increased in March in the anadromous salmon (and were 60% higher than landlocked) and then declined, whereas in landlocked salmon, they increased progressively from January to June (Fig. 3c). Pituitary *pc1* and *pc2* mRNA levels did not significantly differ between strains (Fig. 3d,e). In both anadromous and landlocked salmon, pituitary *pc1* mRNA levels increased between January and March and then declined (Fig. 3d), while pituitary *pc2* mRNA levels increased progressively from January through June (Fig. 3e).

**Figure 4:**
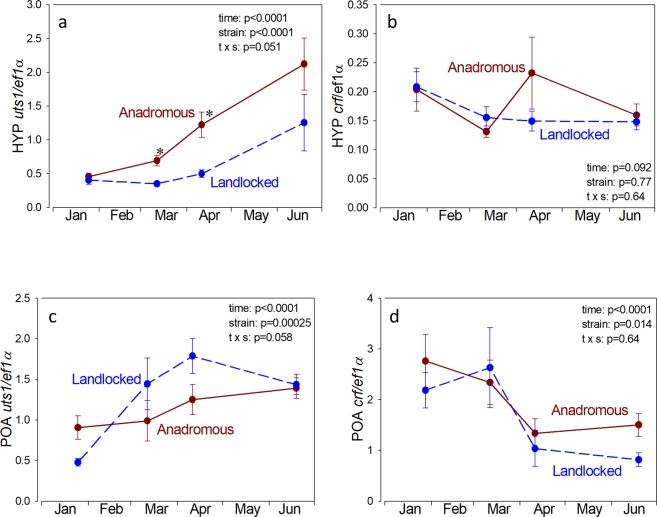
Hypothalamic (HYP) urotensin I (*uts1*; **a**) and corticotropin-releasing factor (*crf*; **b**) and preoptic area (POA) *uts1* (**c**) and *crf* (**d**) mRNA levels in juvenile anadromous and landlocked Atlantic salmon in freshwater. Values are relative to the housekeeping gene *ef1α* and are the mean ± standard error of 8–10 individuals. Results of two-way ANOVA are noted in the figure. Asterisk indicates significant difference from the landlocked strain at the same time period (p < 0.05, Neuman-Keuls test).

Hypothalamic *uts1* mRNA levels were low in January, but increased in the anadromous strain in March and April and were 2.0- and 2.4-fold higher, respectively in anadromous than in landlocked salmon (Fig. 4a). Hypothalamic *crf* mRNA levels did not change significantly over time and did not differ between strains (Fig. 4b). POA *uts1* mRNA levels increased in both groups and were slightly higher in the landlocked than the anadromous salmon (Fig. 4c). POA *crf* mRNA was high in January and decreased in April in both anadromous and landlocked salmon (Fig. 4d).

## Discussion

This study compares key traits related to salinity tolerance, necessary for an anadromous lifecycle, and temporal changes in the neuroendocrine systems implicated in the control of smolt development. The findings demonstrate that salinity tolerance, growth in the first ten days in SW, and the induction of the SW isoform of NKA (NKAα1b) are not upregulated to the same degree in landlocked as in anadromous Atlantic salmon. Hormones that regulate these physiological traits (GH and cortisol) are also stimulated to a lesser degree in the landlocked strain, whereas thyroid hormone and IGF-I profiles are similar in the two strains. To our knowledge this is the first study to show that hypothalamic urotensin I, mRNA levels and plasma ACTH are upregulated during smolt development, and that these components of the hypothalamic-pituitary-interrenal (HPI) axis are stimulated to a greater degree in anadromous smolts. The results indicate that relaxed selection on SW entry over relatively short time scales can result in impacts on the neuroendocrine and endocrine control of life history traits.

It is important to note that while traits associated with SW performance were clearly lower in landlocked than anadromous salmon, there were still increases in the spring. Relaxed selection is predicted to operate more slowly than positive selection^18^ and the development of these traits may not impose significant ‘costs’ (selective disadvantage) to the organisms that would otherwise lead to their complete loss over evolutionary time. Alternatively, pleiotropic actions of GH and cortisol may have maintained their partial upregulation during smolting of landlocked salmon, since in addition to their osmoregulatory actions, GH and cortisol also play a role in the metabolic and behavioral changes that occur during smolting^11^ and these may be important to survival and fitness of both anadromous and landlocked salmon.

The levels of SW tolerance (plasma chloride levels after 24 h exposure to 35 ppt SW) and gill NKA activity are similar in magnitude to what has been observed in a number of other studies of different populations of anadromous Atlantic salmon in mainland North America^19–21^. Peak levels of gill NKA activity of 6 anadromous populations varied between 8 and 12 μmol ADP mg protein^−1^ h^−1^, similar to what was observed for the Connecticut River populations in this study and higher than those achieved in the landlocked Sebago strain. Lower salinity tolerance and gill NKA activity in landlocked salmon as seen in the present study have been observed in several other populations of landlocked salmon^8,16,22,23^, though they were not observed in one landlocked population in Finland^17^. Independent of smolting, larger body size of salmonids confers greater salinity tolerance^24^, so the larger size of landlocked salmon in the present study may have partially obscured an even greater difference in salinity tolerance. The clear differences between strains in gill NKA activity and in the abundance of the SW isoform of NKA (NKAα1b) further support a large difference in SW performance.

Salinity-specific isoforms of the catalytic α-subunit of NKA were first found through molecular approaches in rainbow trout^7^ and then Atlantic salmon^8^, and although they are not universal in euryhaline species, they have been found in other teleost lineages^25,26^. In the present study, there was a 20-fold increase in protein abundance of the NKAα1b in the gill, similar to that reported in previous studies^9^. Gill NKAα1b abundance of landlocked salmon also increased, but to only half the levels seen in anadromous salmon, consistent with previous comparisons at the transcriptional level^8^. Both the temporal increase in gill NKAα1b and the difference in peak abundance levels are similar to the observed differences in SW tolerance. The abundance of the NKAα1a, though somewhat more variable, did not change during smolting or between strains, providing further confirmation that differences in the strains are specific to traits related to SW performance.

Our results demonstrate that there is a clear upregulation of the HPI axis at several levels. CRF and urotensin I are the most potent stimulators of ACTH release in fishes, and CRF is generally regarded as the brain factor that stimulates acute, stress-related release of ACTH and cortisol production^27^. Our results indicate that brain *crf* mRNA levels do not increase in spring and did not differ between anadromous and landlocked salmon. In contrast, both hypothalamic and POA *uts1* mRNA levels increased in spring, and the levels of hypothalamic *uts1* mRNA were significantly higher in anadromous salmon. There were also significant increases in pituitary *pomc-a1*, *pomc-b*, *pc1*, and *pc2* in spring and significantly higher levels of *pomc-b* in anadromous salmon early in spring. It should be noted that these measurements represent a measure of transcription and do not necessarily measure hormone release, especially for pituitary hormones which are known to be stored in cells prior to release. mRNA levels of brain peptides are more likely to be indicative of release, and our results support a critical role for urotensin I in stimulation of the HPI axis during smolting, which in turn stimulates transcription of POMC and the production and release of ACTH by the pituitary, resulting in the observed increase in circulating ACTH and cortisol. Changes in brain urotensin I have previously been implicated in the seasonal control of the HPI axis during spawning of salmon^28^.

Plasma GH levels were substantially higher in anadromous than landlocked salmon throughout spring. There is an important interaction of GH and cortisol axes that includes increased responsiveness of interrenal cortisol production to ACTH^29^ and greater abundance of gill cortisol receptors^30^ following exogenous GH treatment. While GH differed substantially between landlocked and anadromous salmon, there were no significant differences in plasma IGF-I levels. This is curious, as it is known that exogenous IGF-I can promote salinity tolerance. While there is increased local (e.g. gill) production of IGF-I during smolting, partial common garden comparisons of landlocked and anadromous salmon in Norway did not find significant differences in gill IGF-I mRNA levels^31^. We therefore hypothesize that most of the differences in salinity tolerance in anadromous and landlocked salmon are driven by the HPI axis and its interaction with circulating GH levels.

Thyroid hormones increase during smolt development and are thought to control some of the morphological changes such as silvering^11^ and imprinting on stream odorants^32^. As aspects of silvering and imprinting occur in both landlocked and anadromous salmon, we predicted that circulating thyroid hormones would not differ in the two strains. This prediction was largely confirmed; both plasma T_4_ and T_3_ increased in both strains and peak levels of each were actually higher in the landlocked strain. These data support the idea that selection can act on specific endocrine components of a complex developmental process, and that in this case, the thyroid axis has maintained its function while the HPI and GH axes have been altered by relaxed selection on SW performance.

Based on geological data, the Sebago Lake water shed was at or below sea level when the late Wisconsinan Laurentide ice sheet started retreating 15,000–14,000 yr BP, and it subsequently became a FW lake during the rapid postglacial uplift which began between 14,000 and 13,300 yr BP^33^. At present, the Sebago Lake surface is at 82 m above sea level. Although speculative, it appears likely that during this early post-glacial isostatic rebound some waterfall(s) down-river from the lake became impassable for anadromous salmon migration, causing Atlantic salmon in Sebago Lake to become landlocked. The strains of anadromous and landlocked Atlantic salmon used in the present study originated from wild stocks but have had some hatchery intervention. We expect that this has had minimal impact on the genetic underpinning of these traits. Both strains had only one generation reared in freshwater before they were replaced or supplemented each year by wild returning adults, and spawning protocols were designed to maintain genetic diversity. If there were any substantial effect, this freshwater rearing is likely to have negatively impacted primarily the anadromous strain, which have been shown to have similar levels of salinity tolerance and gill NKA activity as wild fish^20,34^.

Our common-garden, phenotypic approach has found evidence for genetically-based differences in the hormonal control of life history traits between landlocked and anadromous populations of Atlantic salmon that are likely to have evolved over the last 14,000 years. Landlocked populations are not uncommon among anadromous species, so it will be of value to determine if the same neuroendocrine differences have repeatedly evolved and establish their genomic basis. Examination of recently introduced populations would also allow for examination of the pace at which these evolutionary changes can occur. The current study demonstrates the importance of *in vivo* physiological approaches combined with enzyme, hormone and neuropeptide assessments at the protein and transcriptional levels in order to elucidate evolutionary changes in the regulation of complex developmental and life history traits.

## Materials and Methods

Eggs from landlocked salmon were obtained from the Sebago Lake strain broodstock held at the Bald Hill Fish Culture Station, Newark, VT. This strain originated from eggs of wild caught fish obtained from Sebago Lake in 1999, and which was supplemented periodically by eggs from wild caught adults from Lake Champlain and Lake Sebago (8 of 12 brood years between 1999 and 2010). Anadromous Atlantic salmon eggs were obtained from the Connecticut River broodstock held at the Roger Reed State Fish Hatchery, Palmer, MA. This strain originated primarily from Penobscot River fish from 1974 to 1994 and was subsequently derived solely from returning adults to the Connecticut River supplemented with F1 broodstock^35^. Previous work using neutral genetic markers has established that these two strains are genetically distinct and that the anadromous populations groups with other nearby anadromous populations^36^. Landlocked and anadromous eggs were transported to the Dwight D. Eisenhower National Fish Hatchery in North Chittenden, VT on the day of fertilization (November 7^th^ and 4^th^, 2011 respectively). The two strains were reared side-by-side with well water (8 °C) in vertical tray incubators until hatching. After hatching, fish were transferred to 246 l troughs supplied with heated (11 °C) well water and fed Bio-Oregon’s Bio-Vita starter diet with automatic feeders. Once the fish reached 2.5 cm in mean fork length, they were transferred to 1 m diameter tanks and fed *ad libitum* 4 times daily. On June 12^th^, 2012 both strains were transferred to outdoor raceways supplied with ambient Furnace Brook water.

In October 2012, fish were transferred from the hatchery to the Conte Anadromous Fish Research Center (Turners Falls, MA, USA) and maintained in 1.7 m diameter tanks (3 tanks per strain) supplied with ambient Connecticut River water at a flow rate of 4 l min^−1^ and provided with continuous aeration. They were maintained under natural photoperiod conditions and fed to satiation (Zeigler Bros, Gardners, PA, USA) using automatic feeders. On January 7^th^, the temperature of all tanks was gradually increased from ambient (3 °C) to 10 ± 1 °C over three days where it remained for the duration of the experiment. This rearing temperature allowed for normal smolt development and direct comparison of seasonal changes that could be directly attributed to smolt development rather than temperature change. All fish sampled were at least 15 cm fork length which is above the established threshold for smolt development in Atlantic salmon^37^. All rearing and sampling were carried in accordance with USGS institutional guidelines and protocol LSC-9096 that was approved by the USGS Leetown Science Center Institutional Animal Care and Use Committee.

Six fish were sampled from each tank on January 28^th^, March 12^th^, April 12^th^, May 9^th^ and June 19^th^ 2013, as described below. An additional 5 fish from each tank were placed into 1-m diameter tanks with 35 ppt SW at 10 °C with particle, biological and charcoal filtration and continuous aeration (SW challenge test) and sampled 24 h later.

On April 19^th^ 2013, 30 fish from each strain were anesthetized with MS-222 (100 mg l^−1^, pH 7.0), implanted with a passive integrated transponder tag and placed into two 1.7 m diameter tanks (each with 15 fish from each strain) at 35 ppt SW with particle, biological and charcoal filtration and continuous aeration. Temperature was maintained at 10 ± 0.5 °C and fish were fed *ad libitum* once daily throughout the growth trial except for April 29^th^, May 14^th^ and May 29^th^, when they were size measured.

Food was withheld for 24 h prior to weighing and/or sampling of fish which occurred between 0900 and 1200 Eastern Standard Time. Fish were anesthetized with MS-222 (200 mg l^−1^, pH 7.0) and blood was drawn by syringe from the caudal vessels and spun at 3200 rcf for 5 min at 4 °C. Plasma was removed and stored at −80 °C. Gill filaments were trimmed from the ceratobranchials and frozen immediately at −80 °C for Western blots. Four to six gill filaments were placed in 100 µl of ice-cold SEI buffer (150 mM sucrose, 10 mM EDTA, 50 mM imidazole, pH 7.3) and frozen at −80 °C for measurement of NKA activity. The brain and pituitary were removed, the hypothalamic region and preoptic area (POA) were dissected according to neuroanatomical landmarks of the salmonid brain as shown in Fig. 1a in Bernier *et al*.^38^, and frozen at −80 °C for mRNA extraction.

### Physiological parameters

NKA activity in gill homogenates was determined using a temperature-regulated microplate method^39^. Ten microliters of samples were run in duplicate in 96-well microplates at 25 °C and read at a wavelength of 340 nm for 10 min on a BioTek Synergy 2 spectrophotometer (BioTek, Winooski, VT). Protein concentration of the homogenate was determined using a BCA protein assay (Thermo Fisher Scientific, Rockford, IL).

Plasma chloride was analyzed by the silver titration method using a Buchler-Cotlove digital chloridometer (LABCONCO, Kansas City, MO, USA) and external standards.

### Western blots

Gill NKAα1a and NKAα1b abundances were quantified by Western immunoblotting as previously outlined^9^ with modification as follows. Gill tissue was homogenized in 10 volumes of SEI plus 0.1% sodium deoxycholate and Complete Mini protease inhibitor tablets (Roche, Indianapolis, IN, USA). The tissue homogenate was centrifuged at 7000 g for 7 min at 4 °C, and 10 µg per lane of supernatant protein was loaded and resolved by SDS-PAGE. Blotted proteins were exposed to primary antibody rabbit anti-NKAα1b at 0.013 μg ml^−1^ and chicken anti-NKAα1a at 0.1 μg ml^−1^. Image J quantified gray values were standardized to the same tissue preparation in every blot to control for blot-to-blot variation.

### Plasma hormones

Plasma cortisol levels were measured by a validated direct competitive enzyme immunoassay as outlined in^40^. Plasma ACTH levels were measured by enzyme linked immunosorbent assay that has previously been validated for use in salmonid plasma^41^. Plasma GH levels were measured by a radioimmunoassay validated for Atlantic salmon^42^. Plasma IGF-I levels were measured by a radioimmunoassay validated for salmonids^43^. Plasma T_4_ and T_3_ concentrations were measured by a direct radioimmunoassay^19^.

### RNA isolation and qPCR

Total RNA was extracted from tissue using TRIzol Reagent according to manufacturer’s instructions (Thermo Scientific, Wilmington, DE, USA). RNA concentration and purity were assessed using a NanoDrop 1000 spectrophotometer (Thermo Scientific). Following extraction, 1 μg of total RNA was DNase treated (Ambion DNase I, Thermo Scientific) and reverse transcribed to cDNA using Superscript II RNaseH^−^ Reverse Transcriptase (Invitrogen, Thermo Scientific). Relative mRNA levels of pituitary pro-opiomelanocortin (*pomc*)*a1*, *a2* and *b*, prohormone convertase (*pc*) *1* and *2*, hypothalamic corticotropin-releasing factor (*crf*) and urotensin I (*uts1*), and POA *crf* and *uts1* were determined by qRT-PCR using a CFX96 system (BioRad, Hercules, CA, USA) with Quanta Perfecta Supermix (Quanta Biosciences; Gaithersburg, MD, USA) and the primers listed in electronic supplementary material, Table S1. The 20 µl reactions contained 10 µl of 2× master mix, 5 µl of diluted cDNA template or no-RT controls, and 2.5 µl of both forward and reverse primers (0.4 µM). Default cycling conditions were used and followed by a melting curve analysis to verify the specificity of each PCR product. Only samples with a unimodal dissociation curve and the predicted melting point temperature were analyzed. To account for differences in amplification efficiency, standard curves were constructed for each gene using known dilutions of cDNA. Input values for each gene were obtained by fitting the average threshold cycle (C_t_) value to the antilog of the gene-specific standard curve, thereby correcting for differences in primer amplification efficiency. To correct for minor variations in template input and transcriptional efficiency, the input values were normalized to the housekeeping gene elongation factor 1α (*ef1α*). Note that the expression of *ef1α* did not change over time or differ between the landlocked and anadromous fish. Finally, non-reverse transcribed RNA and water controls were run to ensure that no genomic DNA was being amplified and the reagents were not contaminated.

### Statistics

Differences between anadromous and landlocked salmon over time were compared using two-way ANOVA. When differences were significant, Student-Newman-Keuls post-hoc comparisons were used to compare them at each time point. mRNA levels did not conform to the assumption of equal variance, so these data were ranked prior to analysis with two-way ANOVA.

## Electronic supplementary material

Supplementary Information

## Data Availability

Original data sets will be made available in Dryad (https://datadryad.org/) upon acceptance of the manuscript.

## References

[1] Crespi EJ, Williams TD, Jessop TS, Delehanty B (2015). Life history and the ecology of stress: how do glucocorticoid hormones influence life-history variation in animals?. Funct. Ecol..

[2] Dantzer B, Swanson EM (2012). Mediation of vertebrate life histories via insulin-like growth factor-1. Biol.l Rev..

[3] Wingfield JC (2015). Coping with change: A framework for environmental signals and how neuroendocrine pathways might respond. Front. Neuroend..

[4] Pigliucci, M. *Phenotypic Plasticity*, Johns Hopkins University Press (2001).

[5] Denver RJ (2009). Comparative endocrinology in the 21st century. Integr. Comp. Biol..

[6] Evans DH, Piermarini PM, Choe KP (2005). The multifunctional fish gill: Dominant site of gas exchange, osmoregulation, acid-base regulation, and excretion of nitrogenous waste. Physiolog. Rev..

[7] Richards JG, Semple JW, Bystriansky JS, Schulte PM (2003). Na+/K+ -ATPase a-isoform switching in gills of rainbow trout (*Oncorhynchus mykiss*) during salinity transfer. J. Exp. Biol..

[8] Nilsen TO (2007). Differential expression of gill Na^+^, K^+^-ATPase a- and b-subunits, Na^+^, K^+^, 2Cl^−^ cotransporter and CFTR anion channel in juvenile anadromous and landlocked Atlantic salmon *Salmo salar*. J. Exp. Biol..

[9] McCormick SD, Regish AM, Christensen AK, Björnsson B (2013). Th. Differential regulation of sodium-potassium pump isoforms during smolt development and seawater exposure of Atlantic salmon. J. Exp. Biol..

[10] Bern, H. A. Endocrinological studies on normal and abnormal salmon smoltification. In *Comparative Endocrinology* (eds Gaillard, P. J. & Boer, H. H.) 77–100, (Elsevier/North Holland Biomedical Press 1978).

[11] McCormick, S. D. Smolt physiology and endocrinology. In *Euryhaline Fishes* (eds McCormick, S. D., Farrell, A. P. & Brauner, C. J.) 199–251, (Academic Press 2013).

[12] Nilsson J (2001). Matrilinear phylogeography of Atlantic salmon (*Salmo salar* L.) in Europe and postglacial colonization of the Baltic Sea area. Molec. Ecol..

[13] Verspoor, E. The evolution of genetic divergence at protein coding loci among anadromos and nonanadromous populations of Atlanic salmon *Salmo salar*. In *Genetics and Evolution of Aquatic Organisms* (ed. Beaumont, A.) 52–67, (Chapman & Hall 1999).

[14] King TL, Kalinowski ST, Schill WB, Spidle AP, Lubinski BA (2001). Population structure of Atlantic salmon (*Salmo salar* L.): a range-wide perspective from microsatellite DNA variation. Molec. Ecol..

[15] Boucher, D. P. & Warner, K. Maine Landlocked Salmon: Life History, Ecology and Management. 1–113, (Maine Department of Inland Fisheries and Wildlife 2006).

[16] Birt TP, Green JM, Davidson WS (1991). Contrasts in development and smolting of genetically distinct sympatric anadromous and nonanadromous Atlantic salmon. Salmo salar. Can. J. Zool..

[17] Piironen J, Kiiskinen P, Huuskonen H, Heikura-Ovaskainen M, Vornanen M (2013). Comparison of smoltification in Atlantic salmon (Salmo salar) from anadromous and landlocked populations under common garden conditions. Ann. Zool. Fenn..

[18] Lahti DC (2009). Relaxed selection in the wild. Trends Ecol. Evol..

[19] McCormick SD (2013). Physiological and endocrine changes in Atlantic salmon smolts during hatchery rearing, downstream migration, and ocean entry. Can. J. Fish. Aquat. Sci..

[20] McCormick SD, Cunjak RA, Dempson B, O’Dea MF, Carey J (1999). Temperature-related loss of smolt characteristics in Atlantic salmon in the wild. Can. J. Fish. Aquat. Sci.

[21] Kelly JT (2015). Evidence for episodic acidification effects on migrating Atlantic salmon Salmo salar smolts. J. Fish Biol..

[22] Nilsen TO, Ebbesson LOE, Stefansson SO (2003). Smolting in anadromous and landlocked strains of Atlantic salmon (Salmo salar). Aquacult..

[23] Foote CJ, Wood CC, Clarke WC, Blackburn J (1992). Circannual cycle of seawater adaptability in *Oncorhynchus nerka*: genetic differences between sympatric sockeye salmon and kokanee. Can. J. Fish. Aquat. Sci..

[24] Parry G (1958). Size and osmoregulation in salmonid fishes. Nature.

[25] Tipsmark. CK (2011). Switching of Na(+), K(+) -ATPase isoforms by salinity and prolactin in the gill of a cichlid fish. J. Endocrinol..

[26] Velotta JP (2017). Transcriptomic imprints of adaptation to fresh water: parallel evolution of osmoregulatory gene expression in the Alewife. Molec. Ecol..

[27] Bernier, N. J., Flik, G. & Klaren, P. H. M. Regulation and contribution of the corticotropic, melanotropic and thyrotropic axes to the stress response in fishes. In *Fish Neuroendocrinology*, *Fish Physiology Vol*. *28* (eds Bernier, N. J., Van der Kraak, G., Farrell, A. P. & Brauner, C. J.) 235–311, (Academic Press 2009).

[28] Westring CG (2008). Seasonal changes in CRF-I and urotensin I transcript levels in masu salmon: Correlation with cortisol secretion during spawning. Gen. Comp. Endocrinol..

[29] Young G (1988). Enhanced response of the interrenal of coho salmon (*Oncorhynchus kisutch*) to ACTH after growth hormone treatment *in vivo* and *in vitro*. Gen. Comp. Endocrinol..

[30] Shrimpton JM, McCormick SD (1998). Regulation of gill cytosolic corticosteroid receptors in juvenile Atlantic salmon: Interaction effects of growth hormone with prolactin and triiodothyronine. Gen. Comp. Endocrinol..

[31] Nilsen TO (2008). Endocrine systems in juvenile anadromous and landlocked Atlantic salmon (*Salmo salar*): Seasonal development and seawater acclimation. Gen. Comp. Endocrinol..

[32] Dittman AH, Quinn TP (1996). Homing in Pacific salmon: Mechanisms and ecological basis. J. Exp. Biol..

[33] Koteff C, Robinson GR, Goldsmith R, Thompson WB (1993). Postglacial uplift and synglacial sea levels in coastal central New England. Quatern. Res..

[34] McCormick SD, Björnsson B (1994). Th. Physiological and hormonal differences among Atlantic salmon parr and smolts reared in the wild, and hatchery smolts. Aquacult..

[35] Gephard S, McMenemy J (2004). An overview of the program to restore Atlantic salmon and other diadromous fishes to the Connecticut river with notes on the current status of these species in the river. American Fisheries Society Monograph.

[36] Spidle AP (2003). Population structure of Atlantic salmon in Maine with reference to populations from Atlantic Canada. Trans. Amer. Fish. Soc..

[37] McCormick SD, Shrimpton JM, Moriyama S, Björnsson B (2007). Th. Differential hormonal responses of Atlantic salmon parr and smolt to increased daylength: A possible developmental basis for smolting. Aquacult..

[38] Bernier NJ, Alderman SL, Bristow EN (2008). Heads or tails? Stressor-specific expression of corticotropin-releasing factor and urotensin I in the preoptic area and caudal neurosecretory system of rainbow trout. J. Endocrinol..

[39] McCormick SD (1993). Methods for non-lethal gill biopsy and measurement of Na+, K+ -ATPase activity. Can. J. Fish. Aquat. Sci..

[40] Carey JB, McCormick SD (1998). Atlantic salmon smolts are more responsive to an acute handling and confinement stress than parr. Aquacult..

[41] Craig PM, Haider A-T, Bernier NJ (2005). Differential Increase in Forebrain and Caudal Neurosecretory System Corticotropin-Releasing Factor and Urotensin I Gene Expression Associated with Seawater Transfer in Rainbow Trout. Endocrinology.

[42] Björnsson B (1994). The interrelation between photoperiod, growth hormone, and sexual maturation of adult atlantic salmon (*Salmo salar*). Gen. Comp. Endocrinol..

[43] Moriyama S (1994). Development of a homologous radioimmunoassay for coho salmon insulin-like growth factor-I. Gen. Comp. Endocrinol..

