# Author Correction: The evolutionary consequences for seawater performance and its hormonal control when anadromous Atlantic salmon become landlocked

**DOI:** 10.1038/s41598-019-55962-6

**Published:** 2019-12-24

**Authors:** Stephen D. McCormick, Amy M. Regish, William R. Ardren, Björn Thrandur Björnsson, Nicholas J. Bernier

**Affiliations:** 1 U.S. Geological Survey, Leetown Science Center, Conte Anadromous Fish Research Laboratory, Turners Falls, MA 01376 USA; 2 U.S. Fish and Wildlife Service, Lake Champlain Fish and Wildlife Conservation Office, 11 Lincoln Street, Essex Junction, VT 05452 USA; 3 Fish Endocrinology Laboratory, Department of Biological and Environmental Sciences, University of Gothenburg, S40530 Gothenburg, Sweden; 4 Department of Integrative Biology, University of Guelph, Ontario, N1G 2W1 Canada

Correction to: *Scientific Reports* 10.1038/s41598-018-37608-1, published online 30 January 2019

A supplementary file containing Table S1 was omitted from the original version of this Article. This has been corrected in the HTML and PDF versions of the Article.

