# Supplementary material for: The evolutionary consequences for seawater performance and its hormonal control when anadromous Atlantic salmon become landlocked
Source: Sci Rep. 2019 Jan 30;9:968. doi: 10.1038/s41598-018-37608-1 (PMC6353943; doi:10.1038/s41598-018-37608-1)
Supplement: Supplementary file 1 — Supplementary Information [file 41598_2018_37608_MOESM1_ESM.docx]

McCormick S.D., Regish A.M., Ardren W.R., Björnsson B.T. and Bernier N.J. (2019). The evolutionary consequences for seawater performance and its hormonal control when anadromous Atlantic salmon become landlocked. Scientific Reports 9:968. https://doi.org/10.1038/s41598-018-37608-1

Table S1. Nucleotide sequences of Atlantic salmon primers used for qRT-PCR

| Gene | Accession no. | Efficiency | Sequence (5’-3’) |
| --- | --- | --- | --- |
| *crf* | DY733166 | 96.4% | F: ACAACGACTCAACTGAAGATCTCG  R: GAGTAAATTGAGCTTCATGTCAGG |
| *ef1α* | BG933853 | 94.5% | F: TGCCCCTCCAGGATGTCTAC  R: CACGGCCCACAGGTACTGT |
| *pc1* | [XM014132048](https://www.ncbi.nlm.nih.gov/nucleotide/XM_014132048?report=genbank&log$=nuclalign&blast_rank=1&RID=54XGAP6R014) | 97.0% | F: TCAATGACAACGACCCTGAC  R: ACCTGGTTCCATGCTTGTTC |
| *pc2* | BT045124 | 98.7% | F: GGACCCAGAGAGCTCACTTT  R: CTCCAGAGGCCCATACGTAG |
| *pomc-a1* | AB462418 | 97.7% | F: TGGAAGGGGGAGAGGGAGAG  R: CGTCCCAGCTCTTCATGAAC |
| *pomc-a2* | AB462419 | 100.1% | F: CTGGAGGCTGGGACTGCGGA  R: CGTCCCAGCTCTTCATGAAC |
| *pomc-b* | DQ508935 | 99.7% | F: GACTAAGGTAGTCCCCAGAACCCTCAC  R: GACAGCGGTTGGGCTACCCCAGCGG |
| *uts1* | XM014205273 | 95.3% | F: AGGAGACAAAGTACTGGGCA  R: AGGCTTCATAGTGCTGGACA |

*crf*, corticotropin-releasing factor; *ef1α*, elongation factor 1α; F, forward; *pc*, prohormone convertase; *pomc*, pro-opiomelanocortin; R, reverse; *uts1*, urotensin I.
